# In Vitro Experiments on the Effects of GP-2250 on BRAF-Mutated Melanoma Cell Lines and Benign Melanocytes

**DOI:** 10.3390/ijms242015336

**Published:** 2023-10-19

**Authors:** Thilo Gambichler, Friederike Harnischfeger, Marina Skrygan, Britta Majchrzak-Stiller, Marie Buchholz, Thomas Müller, Chris Braumann

**Affiliations:** 1 Skin Cancer Center Ruhr-University, Department of Dermatology, Venereology and Allergology, Ruhr-University Bochum, 44791 Bochum, Germany; friederike.harnischfeger@rub.de (F.H.); m.skrygan@klinikum-bochum.de (M.S.); 2 Department of General and Visceral Surgery, Division of Molecular and Clinical Research, St. Josef-Hospital, Ruhr-University Bochum, 44791 Bochum, Germany; britta.majchrzak@rub.de (B.M.-S.); marie.buchholz@klinikum-bochum.de (M.B.); 3 Geistlich Pharma AG, 6110 Wolhusen, Switzerland; thomas.mueller@geistlich.com; 4 Department of General, Visceral and Vascular Surgery, Evangelisches Klinikum Gelsenkirchen, Akademisches Lehrkrankenhaus der Universität Duisburg-Essen, 45878 Gelsenkirchen, Germany; chris.braumann@evk-ge.de; 5 Department of General, Visceral and Tumor Surgery, Evangelisches Klinikum Herne, Akademisches Lehrkrankenhaus der Ruhr-Universität Bochum, 44623 Herne, Germany

**Keywords:** cutaneous melanoma, GAPDH inhibitors, resistance, skin cancer, PI3K/AKT/mTOR, AKT, STAT3, targeted therapy, glycolysis

## Abstract

Enhanced glycolysis (Warburg effect) driven by the BRAF oncogene, dysregulated GAPDH expression, and activation of the PI3K/AKT/mTOR signaling pathway may significantly contribute to the resistance-targeted therapy of BRAF-mutated melanomas. Therefore, we aimed to study for the first time the anti-tumor activity of the GAPDH inhibitor GP-2250 in BRAF-mutated melanoma cell lines and benign melanocytes. We employed three melanoma cell lines and one primary melanocyte cell line (Ma-Mel-61a, Ma-Mel-86a, SH-4 and ATCC-PCS-200-013, respectively), which were exposed to different GP-2250 doses. GP-2250’s effects on cell proliferation and viability were evaluated by means of the BrdU and MTT assays, respectively. The RealTime-Glo Annexin V Apoptosis and Necrosis Assay was performed for the evaluation of apoptosis and necrosis induction. RT-PCR and western blotting were implemented for the determination of AKT and STAT3 gene and protein expression analyses, respectively. The melanoma cell lines showed a dose-dependent response to GP-2250 during BrDU and MTT testing. The RealTime-Glo Annexin V assay revealed the heterogenous impact of GP-2250 on apoptosis as well as necrosis. With respect to the melanoma cell lines Ma-Mel-86a and SH-4, the responses and dosages were comparable to those used for the MTT viability assay. Using the same dose range of GP-2250 administered to melanoma cells, however, we observed neither the noteworthy apoptosis nor necrosis of GP-2250-treated benign melanocytes. The gene expression profiles in the melanoma cell lines for AKT and STAT3 were heterogenous, whereby AKT as well as STAT3 gene expression were most effectively downregulated using the highest GP-2250 doses. Immunoblotting revealed that there was a time-dependent decrease in protein expression at the highest GP-2250 dose used, whereas a time- as well as dose-dependent AKT decrease was predominantly observed in Ma-Mel-61a. The STAT3 protein expression of Ma-Mel-86a and SH-4 was reduced in a time-dependent pattern at lower and moderate doses. STAT3 expression in Ma-Me-61a was barely altered by GP-2250. In conclusion, GP-2250 has anti-neoplastic effects in BRAF-mutated melanoma cell lines regarding tumor cell viability, proliferation, and apoptosis/necrosis. GP-2250 is able to downregulate the gene and protein expression of aberrant tumorigenic pathways in melanoma cell lines. Since GP-2250 is a GAPDH inhibitor, the substance may be a promising combination therapy for tumors presenting the Warburg effect, such as melanoma.

## 1. Introduction

Mutations in BRAF, particularly the V600E, are frequently harbored in patients with cutaneous melanoma. These genetic alterations are considered to be one of the main drivers of the malignant transformation of melanocytes, even though we and other research groups have shown that these mutations are also very frequently found in benign melanocytic nevi [1,2,3]. Currently, the optimal treatment scenario for advanced melanoma patients depends on the mutation status of BRAF. With the absence of BRAF mutations, the patients have only the treatment option using immune checkpoint inhibitors as state-of-the-art management. In the presence of BRAF mutations, patients have a second highly effective option, which includes targeted therapies with BRAF inhibitors (BRAFi) in combination with MEK inhibitors (MEKi) [1,2]. However, although this approach has considerably improved the prognosis for patients with metastatic melanoma, 30–40% of patients do not respond to targeted therapy or relapse during treatment due to primary (intrinsic) and secondary (acquired) resistance, respectively. For example, primary resistance occurs when patients do not respond to the initial therapy, which can be explained by the activation of substitutive or bypass pathways such as the upregulation of the PI3K/AKT/mTOR pathway [4,5,6,7,8]. Hence, the combination of BRAFi/MEKi and inhibitors of the PI3K/AKT/mTOR pathway may overcome the issue of resistance development [7,8,9,10,11,12].

**Figure 1:**
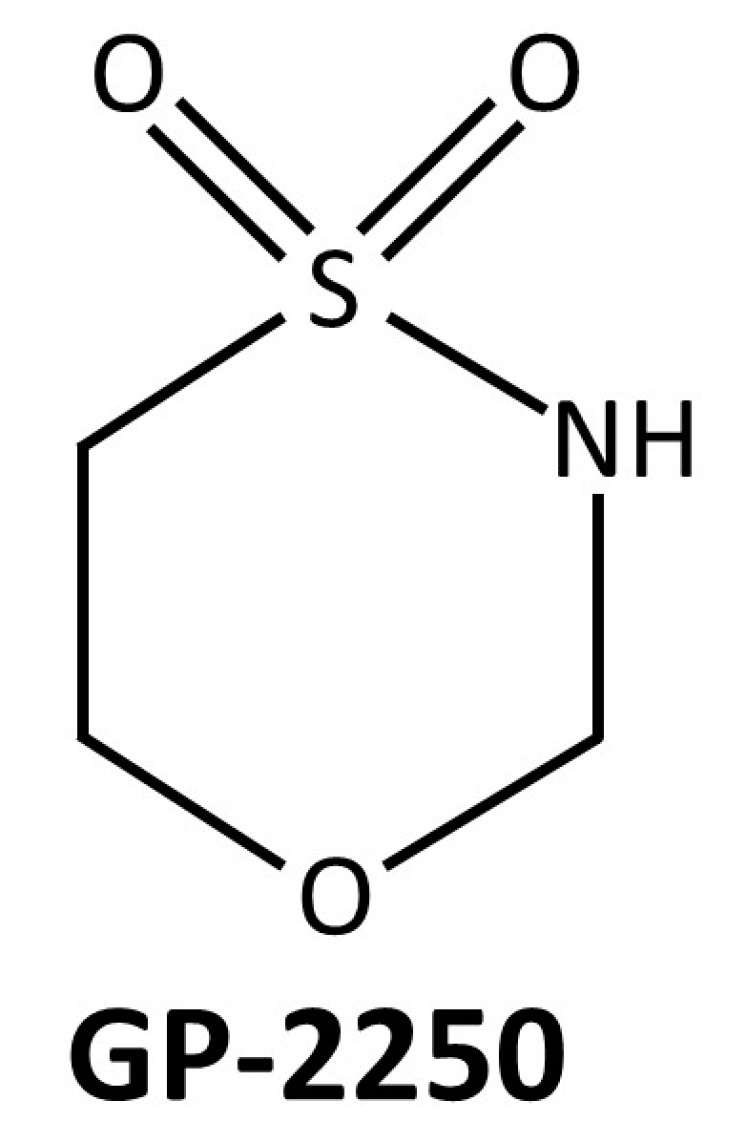
Molecular structure of the substance GP-2250, which is an oxathiazinane derivative.

GP-2250 (Figure 1) is a relatively novel anti-neoplastic substance that has recently been investigated in different cancers [13,14,15,16].

The substance is an oxathiazinane (tetrahydro-1,4,5-oxathiazin-4,4-dioxide) and has inhibitory effects on glyceraldehyde 3-phosphate dehydrogenase (GAPDH) expression and activity, selectively resulting in oxidative stress, mitochondrial dysfunction, and programmed cell death in cancer cells [16].

As shown in in vitro as well as in vivo experiments, GP-2250 is able to downregulate the viability of pancreatic carcinoma cells, which is also accompanied by the induction of apoptosis as well as necrosis. The same study group reported on the anti-tumor activity of GP-2250, investigating other malignancies as well [13,14,15,16]. Importantly, the substance proved to be safe in nude mice, indicated by acute or chronic toxicity only at extremely high concentrations (acute toxicity at 2000 mg/kg*BW and chronic toxicity at concentrations higher than 1000 mg/kg*BW in nude mice). No changes in body weight or vital function were observed at lower concentrations [13]. Thus, in a phase I/II trial, GP-2250 is currently being investigated in combination with gemcitabine in patients with advanced pancreatic carcinoma (clinicaltrials.gov: NCT03854110) [15]. Moreover, the anti-neoplastic activity of GP-2250 has been investigated in malignant skin cancers, including Merkel cell carcinoma [14].

As reported by Guo and co-workers [17], GAPDH being consistently regarded as the main housekeeping and reference gene/protein for expression quantification in tumors has been questioned and challenged by accumulated evidence. GAPDH is an indispensable enzyme for glucose metabolism, and is markedly upregulated in glycolytic cancer cells. The distinctive feature of GAPDH is that its catalytic function is one of the critical rate-limiting steps of glycolysis [18]. El Sayed [19] recently proposed that in transformed cells and hyper-glycolytic cancer cells, the Warburg effect (permanent conversion of pyruvate to lactate) occurs secondary to a vicious cycle and a closed circuit between GAPDH and lactate dehydrogenase, causing increased endogenous oxidative stress and subsequent carcinogenesis. Melanoma (having low lactate dehydrogenase-A expression) exhibits resistance to MAPK signaling inhibitors, which target the Warburg effect [19]. It has been shown by Hall et al. [20] that dysfunctional oxidative phosphorylation makes melanoma cells addicted to glycolysis, driven by the BRAF (V600E) oncogene. Moreover, Ramos et al. [21] reported that a dysregulated GAPDH expression is observed during melanoma progression. Hence, it appears reasonable to evaluate the effect of GAPDH inhibitors in melanoma.

We aimed to study for the first time the anti-tumor activity of GP-2250 in BRAF-mutated melanoma cell lines and benign melanocytes, also addressing the expression of parts of the PI3K/AKT/mTOR signaling pathway.

## 2. Results

**Figure 2:**
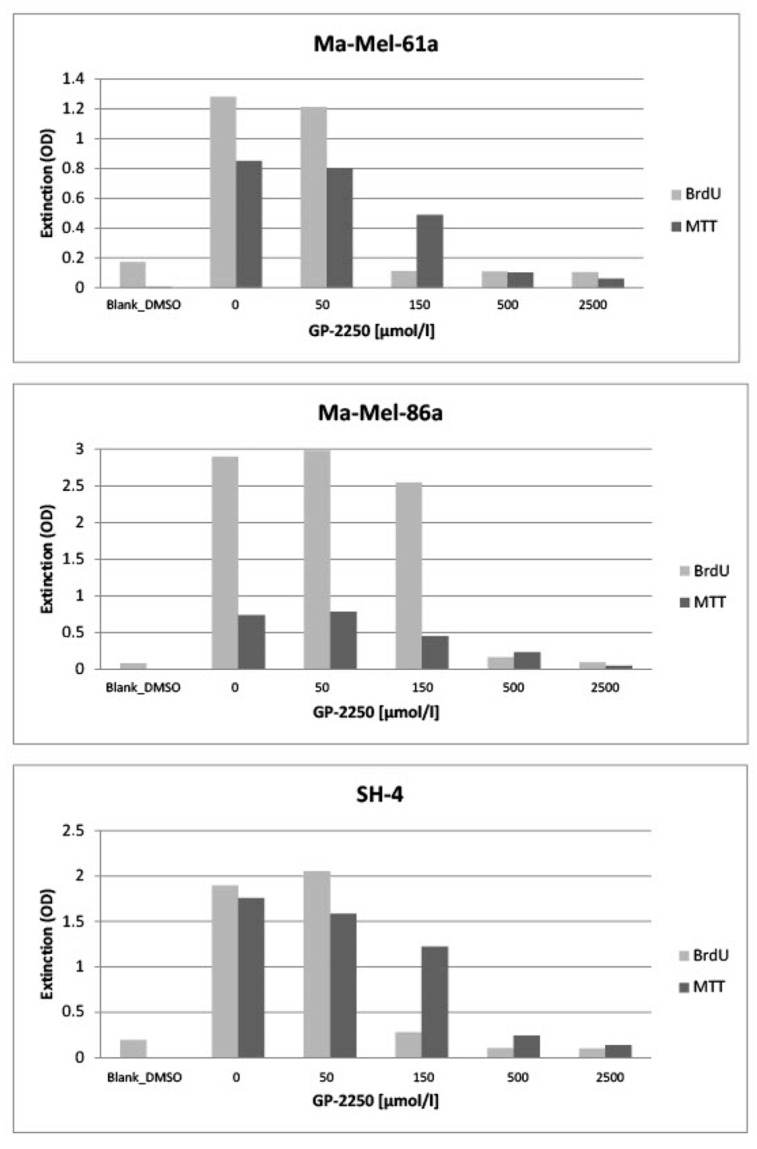
All three melanoma cell lines exhibited a more or less dose-dependent response to GP-2250 during the BrDU proliferation and the MTT viability assays, whereas proliferation as well as viability were drastically reduced by a GP-2250 concentration of 500 µmol/L.

In Figure 2, all three melanoma cell lines (Ma-Mel-61a, Ma-Mel-86a, SH-4) showed a dose-dependent response to GP-2250 during BrDU testing to a different extent, thus demonstrating the anti-proliferative effect of GP-2250 on melanoma cells. Unlike MA-Mel-86a, Ma-Mel-61a and SH-4 exhibited the effective inhibition of cell proliferation at a dosage of 150 µmol/L GP-2250. Figure 2 also illustrates the dose-dependent response to GP-2250 in all three melanoma lines during the MTT viability assay, demonstrating its cytotoxic effects on melanoma cells, which were most obvious at a GP-2250 dosage of 150 µmol/L and higher at onset.

**Figure 3:**
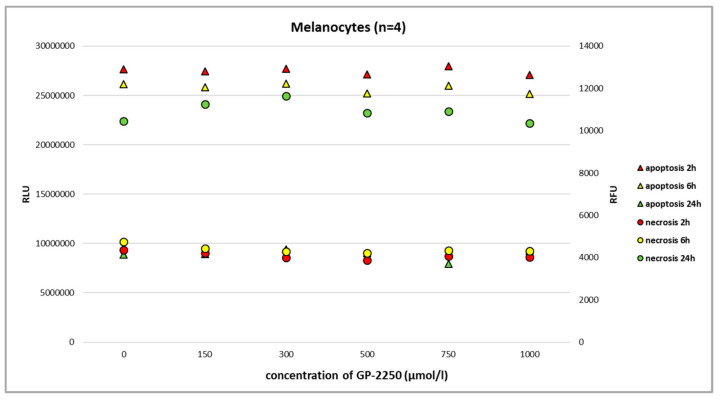
The RealTime-Glo Annexin V Apoptosis and Necrosis Assay (RLU, luminescence; RFU, fluorescence) performed on benign melanocytes treated with different doses of GP-2250. We used a commercially available cell line with normal human primary epidermal melanocytes (ATCC-PCS-200-013, LGC Standards GmbH, Wesel, Germany). The assay revealed that even the highest dosage of GP-2250 did not result in noteworthy apoptosis/necrosis.

**Figure 4:**
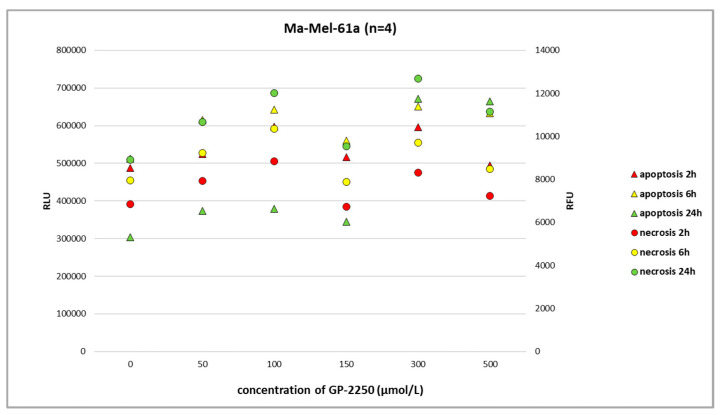
The RealTime-Glo Annexin V Apoptosis and Necrosis Assay (RLU, luminescence; RFU, fluorescence) performed on the melanoma cell line Ma-Mel-61a treated with different doses of GP-2250. A more (24 h) or less dose-dependent increase in apoptosis and necrosis was apparent.

**Figure 5:**
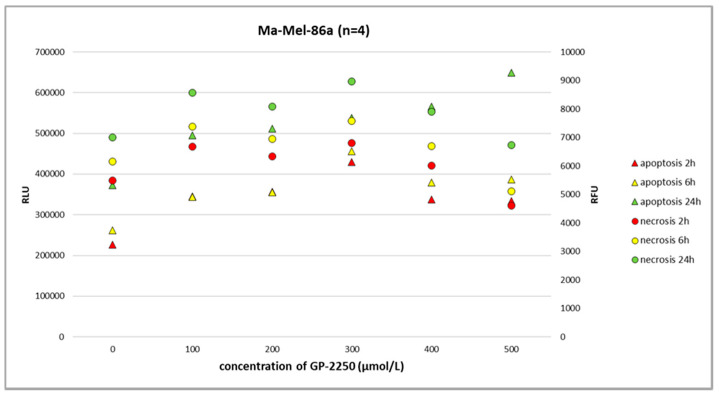
The RealTime-Glo Annexin V Apoptosis and Necrosis Assay (RLU, luminescence; RFU, fluorescence) performed on the melanoma cell line Ma-Mel-86a treated with different doses of GP-2250. The effects of GP-2250 on apoptosis were most evident at a dosage of 500 µmol/L after 24 h.

**Figure 6:**
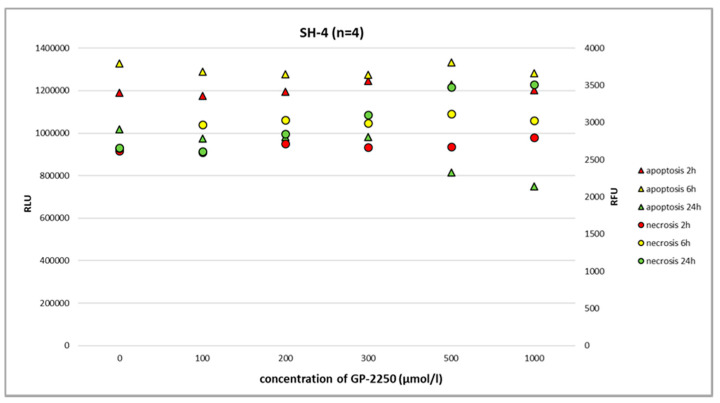
The RealTime-Glo Annexin V Apoptosis and Necrosis Assay (RLU, luminescence; RFU, fluorescence) performed on the melanoma cell line SH-4 treated with different doses of GP-2250. After 24 h, GP-2250 predominantly induced necrosis at a dosage of 500 µmol/L and higher.

Using the RealTime-Glo Annexin V Apoptosis and Necrosis Assay and the full range of GP-2250 dosage, we observed neither the noteworthy apoptosis nor necrosis of benign melanocytes at 2 h, 6 h, and 24 h (Figure 3, Figure 4, Figure 5 and Figure 6).

In melanoma cells, GP-2250 induced programmed cell death when tested after 24 h using the RealTime-Glo Annexin V Apoptosis and Necrosis Assay. The doses of GP-2250 were comparable to those used in the MTT test (150 µmol/L and higher). In the Ma-Mel-61a cell line, GP-2250 induced both apoptosis and necrosis, but in a heterogeneous pattern (Figure 4). As shown in Figure 5, Ma-Mel-86a showed, at a dosage of 500 µmol/L, the induction of apoptosis after 24 h, which was more pronounced than necrosis. Necrosis was predominantly observed in the melanoma cell line SH-4 at a dosage of 500 µmol/L and higher doses (Figure 6).

**Figure 7:**
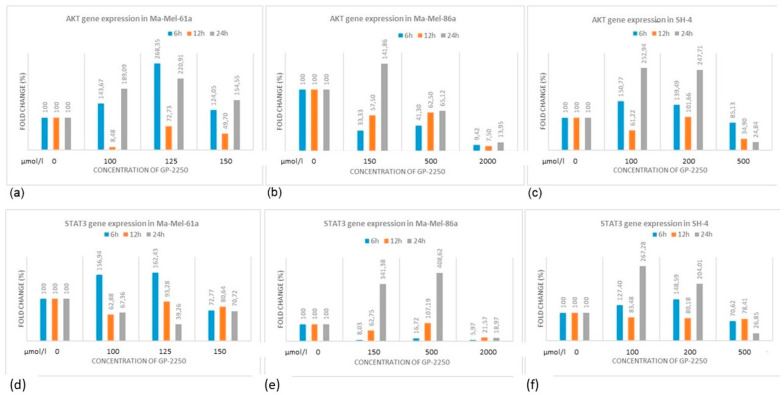
AKT (**a**–**c**) and STAT3 (**d**–**f**) expression profiles in three melanoma cell lines treated with different concentrations of GP-2250.

Using gene expression profiling, we aimed to study the components of the PI3K/AKT/mTOR pathway, which is involved in resistance development in BRAF-mutant melanomas. As shown in Figure 7, the gene expression profiles in melanoma cell lines for AKT and STAT3 were heterogeneous, whereas the downregulation of AKT as well as STAT3 gene expression was most effectively downregulated using GP-2250 doses of 500 µmol/L.

**Figure 8:**
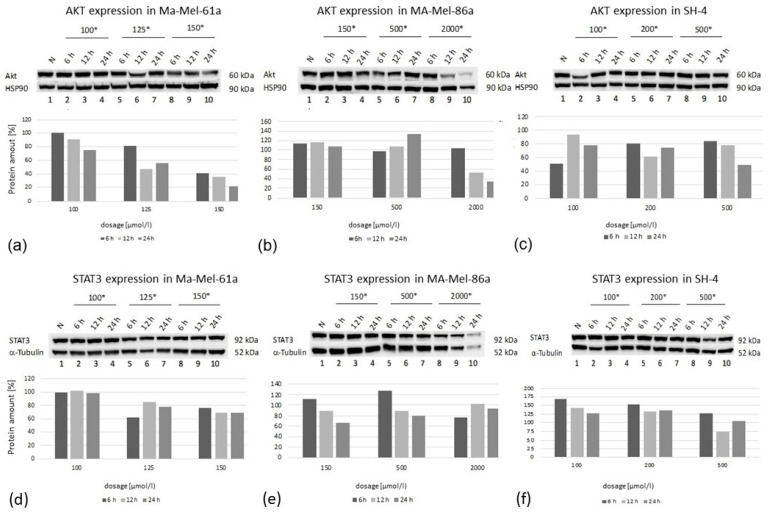
Western blot analysis of AKT (**a**–**c**) and STAT3 (**d**–**f**) protein expression at three time points (6 h, 12 h, 24 h) in three melanoma cell lines after treatment using three individual dosages of GP-2250. HSP-90 was used as the internal control (*, dosage in µmol/L).

Immunoblotting was performed for the protein expression analysis of AKT and STAT3 in all three melanoma cell lines (Figure 8). Cells were exposed to GP-2250 applied in three individual concentrations, having proven effective in preceding experiments.

In all cell lines, there was a time-dependent decrease in protein expression at the highest GP-2250 dose used, whereas a time- as well as dose-dependent AKT decrease was predominantly observed in Ma-Mel-61a. Regarding STAT3 expression, the protein expression of Ma-Mael-86a and SH-4 was reduced in a time- dependent manner at lower and moderate doses (Figure 8). STAT3 expression in Ma-Me-61a was only slightly decreased when moderate and higher GP-2250 dosages were used. Together, AKT and STAT3 protein expression did not consistently match the results obtained from the gene expression analyses.

## 3. Discussion

In the present study, we have shown that all three melanoma cell lines showed a dose-dependent response to GP-2250 during BrdU testing to a different extent, demonstrating the anti-proliferative effect of the substance on melanoma cells. Moreover, a dose-dependent response to GP-2250 was found in all three melanoma lines during the MTT viability assay, demonstrating its cytotoxic effects on melanoma cells. The RealTime-Glo Annexin V Apoptosis and Necrosis Assay revealed the heterogeneous impact of GP-2250 on apoptosis as well as necrosis. With respect to the melanoma cell lines Ma-Mel-86a and SH-4, the responses and dosages were comparable to those used for the MTT viability assay previously. Using the same dose range of GP-2250 administered to melanoma cells, however, we observed neither the noteworthy apoptosis nor necrosis of GP-2250-treated benign melanocytes at 2 h, 6 h, and 24 h. This result may indicate that normal melanocytes are much less prone to GAPDH-inhibiting effects than malignant melanoma, particularly GAPDH, which is not rate limiting in normal cells [18,22]. Naturally, melanocytes are located in a mildly hypoxic environment with an O_2_ concentration of about 10%, which may predispose melanoma to stronger hypoxia with O_2_ concentrations smaller than 1%. Hence, we speculate that GP-2250 might induce apoptosis and necrosis in tumor cells with high GPDH activity and strongly hypoxic conditions [18,22,23].

We exclusively studied cell lines with BRAF (V600E) mutation. Importantly, BRAF mutations in melanoma affect metabolism via the hypoxia-inducible factor 1 (HIF-1), which is the key oxygen sensor and principal regulator of hypoxia-mediated gene response, along with HIF-2. BRAF (V600E) mutation was found to directly regulate HIF-1 expression in cutaneous melanoma [18,24]. Moreover, the increase in HIF-1α induced by BRAF (V600E) mutation might significantly contribute to melanoma genesis in association with the PI3K/AKT/mTOR pathway, which is known to play a relevant role in early melanoma as well as resistance to BRAFi/MEKi therapy [25]. Indeed, the fast and significant efficacy of BRAFi/MEKi therapy is frequently temporary. Resistance is observed in most cases and develops through a variety of cellular mechanisms. Patel et al. [6] and others have demonstrated that elevated levels of reactive oxygen species (ROS) are a mediator of resistance to BRAFi/MEKi in melanoma. ROS levels are increased in acute and chronic therapy with these inhibitors [6]. Moreover, the PI3K/AKT/mTOR pathway is also subjected to redox regulation in the presence of excess ROS [6]. PI3K/AKT/mTOR signaling is a regulator of cellular functions implemented by numerous downstream effectors, including the activation of the JAK2/STAT3 signaling pathway via phosphorylating STAT3. JAK2/STAT3 belongs to non-receptor tyrosine kinases, playing a crucial role in the proliferation and apoptosis of tumor cells [26]. Importantly, Pópulo et al. [5] recently reported that the mitogen-activated protein kinases (MAPK) and PI3K/AKT/mTOR signaling is dysregulated and interconnected in melanoma, and that the presence of the Warburg effect in melanoma cells can alter the response to BRAFi/MEKi. Apart from the activation of the aforementioned pathways, metabolic dysregulations play an important role in melanoma pathobiology as well. As mentioned above, melanoma presents the Warburg effect, accompanied by enhanced glycolysis, which does not depend on the oxygen level. However, the Warburg effect may be reversed by the metabolic modulators, including konningic acid, dimethyl fumarate, and perhaps GP-2250 [18,27].

By contrast, the gene expression profiles in the melanoma cell lines for AKT and STAT3 were heterogeneous, whereas the downregulation of AKT as well as STAT3 gene expression was most effectively downregulated using the highest GP-2250 doses. Western blotting revealed that in all cell lines investigated, there was a time-dependent decrease in protein expression at the highest GP-2250 dose used, whereas a time- as well as dose-dependent AKT decrease was predominantly observed in Ma-Mel-61a. The STAT3 protein expression of Ma-Mel-86a and SH-4 was reduced in a time-dependent pattern at lower and moderate doses. STAT3 expression in Ma-Me-61a was barely altered by GP-2250. The discrepancies between the gene and protein expression analyses observed may be due to relatively short time intervals of measurements. It is possible that the measurements at 36 h and 48 h had resulted in more consistent protein and gene analyses. As discussed by Gavish et al. [28], the comparison of global cell states across tumors underscores a high degree of cellular heterogeneity that remains difficult to interpret. Nevertheless, our data indicate the downregulating effect of GP-2250 on proliferation, cell viability, apoptosis/necrosis, and AKT/STAT3 signaling in melanoma cells. Hence, a combination of BRAFi/MEKi and GP-2250 may result in the decreased occurrence of resistance via the downregulation of AKT/STAT3 signaling and by counteracting the Warburg effect. Indeed, Tran et al. [7] and others have shown that the combination of MAPK and PI3K/AKT/mTOR inhibitors may sensitize melanoma cells to therapy and overcome resistance. Even though the tested concentrations of the substance do not seem to be small, the dosage for chronic and acute toxicity in mice showed the very low toxicity profile of substance GP-2250 [9], which is currently being studied in a clinical phase I/II trial for patients with pancreatic cancer [15]. The results of the clinical trial will additionally indicate possibilities for the application of GP-2250 in combination with BRAFi/MEKi in patients with melanoma, for example.

## 4. Materials and Methods

### 4.1. Cell Lines and Cultivation Methods

Three human BRAF-mutated CM cell lines were studied in this work: Ma-Mel-61a (source: RRID:CVCL_C291), Ma-Mel-86a (source: RRID:CVCL_A221), and SH-4 (source: RRID:CVCL_1692). All cell lines originated from CM metastases. Based on the material transfer agreement, the cell lines have been previously provided to J.C. Becker by D. Schadendorf (Department of Dermatology, University Duisburg-Essen, Essen, Germany). As a control, we used a commercially available cell line with normal human primary epidermal melanocytes (ATCC-PCS-200-013, LGC Standards GmbH, Wesel, Germany). The cell lines MaMel61a and MaMel86a were maintained in RPMI-1640, supplemented with 10% Fetal Bovine Serum (FBS) and Penicillin/Streptomycin, each at 100 U/mL (PAN Biotech GmbH, Aidenbach, Germany). Cell lines SH4 were maintained in DMEM (LGC Standards GmbH) supplemented with 10% Fetal Bovine Serum (FBS) and Penicillin/Streptomycin, each at 100 U/mL. The culture media for melanocytes was Dermal Cell Basal Medium additionally supplemented with Adult Melanocyte Growth Mix (LGC Standards GmbH). All cells were maintained as a monolayer at 37 °C with 5% CO_2_ in a humidified atmosphere to 60–80% confluency.

### 4.2. Reagents

Powdered GP-2250 (kindly provided by Geistlich Pharma AG, Wolhusen, Switzerland) was stored at room temperature and freshly prepared every two weeks by dissolving it in double-distilled water. It was set to a physiological pH, sterile-filtered and stored, protected from light.

### 4.3. MTT

MTT assays were performed on all three CM cell lines in order to analyze calorimetrically the antineoplastic effect of the substance GP2250. Cells were seeded individually to obtain a sub-confluent monolayer in a 96-well plate format and incubated for 24 h prior to treatment. To examine its dose–response relationship, cells were incubated with individual increasing concentrations (from 50 up to 4500 µmol/L) depending on the cell line and ddH2O as the control for 24 h. Following the stated exposition times, 10 µL of MTT (3-(4,5-Dimethylthiazol-2-yl)-2,5-diphenyltetra-zoliumbromid) reagent (5 mg/mL) was added and incubated for 2 h before violet Formazan crystals were dissolved in 100 µL of DMSO (dimethyl sulfoxide). The viability was analyzed using a microplate absorbance reader (ASYS, UVM340, Anthos Mikrosysteme GmbH, Friesoythe, Germany) by determining the optical density at 560 nm (reference wavelength 720 nm). The assay was performed using 8 replicates in three independent experiments with consecutive passages.

### 4.4. BrdU Proliferation Assay

Cells were seeded individually to obtain a sub-confluent monolayer in a 96-well plate format and incubated for 24 h prior to treatment. In order to examine the dose–response of substance GP-2250 regarding its anti-proliferative activity, cells were incubated with individual increasing concentrations of GP-2250 (from 50 up to 3500 µmol/L) depending on the cell line and with ddH2O as the control for 6 h, and submitted for BrdU proliferation assay (5-bromo-2-deoxyuridine)-ELISA (Roche Applied Science, Mannheim, Germany) according to the manufacturer’s instructions. The incubation time of 6 h was appropriate for the BrdU proliferation assay in previous experiments. Based on the incorporation of the thymidine analogue BrdU during DNA synthesis, the amount of synthesized DNA was detected using a microplate absorbance reader (ASYS, UVM340, Anthos Mikrosysteme GmbH) at 450 nm with a reference wavelength of 550 nm. BrdU assays were performed with 8 replicates of three independent experiments with consecutive passages.

### 4.5. Apoptosis/Necrosis Assay

The cells were seeded in a sterile, white 96-well tissue culture plate at a concentration of 10,000 or 30,000 cells/100 µL volume/well, depending on the cell line. All cell lines were incubated for 24 h at 37 °C and with 5% CO_2_. After 24 h, the supernatant was discarded and the cells with GP-2250 were stimulated at the concentration of 0.0–2000 µmol/L with a total of 100 µL/well. Analysis was performed with RealTime-Glo Annexin V Apoptosis and Necrosis Assay (Promega, Madison, WI, USA) according to the manufacturer’s protocol. This assay measures the exposure of phosphatidylserine on the outer leaflet of the cell membrane during the apoptotic process, and the loss of membrane integrity during secondary necrosis. Annexin V binding is detected with a simple luminescence signal, and necrosis is detected with a fluorescent DNA binding dye. The luminescence signal and the fluorescence signal were measured on the GloMax Discover Detection System GM 3000 + 3030 (Promega) after 2 h, 6 h and 24 h.

### 4.6. PCR

The total RNA was extracted from skin tissue samples using the Maxwell^®^ RSC SimplyRNA Tissue Kit (Promega) according to the manufacturer’s protocol. Quantitative analysis was performed using real-time RT-PCR in accordance with the MIQE Guidelines using the PowerSYBR Green PCR Master Mix on a QuantStudio 5 (Applied Biosystems by Thermo Fisher Scientific, Rockford, IL, USA). Primer Express software, v3.0.1 (PE Applied Biosystems, Foster City, CA, USA) was used to design the PCR primers. Two widely used reference genes, namely ß-2-microglobulin (ß2-MG) and RPL-38, were tested. The latter gene exhibited the most stable expression levels, and this gene was used as the housekeeping gene. Primers were produced by the custom oligonucleotide synthesis service TIB Molbiol (Berlin, Germany). The well-established comparative −ΔΔCT method was used. Target mRNA expression was normalized between test samples based on the corresponding RPL38 mRNA transcript levels in each skin sample.

### 4.7. Western Blot Analysis

Protein isolation was performed using RIPA (Radioimmunoprecipitation assay) lysis (Abcam, Cambridge, UK). Subsequently, a BCA (Bicinchoninic acid) assay (Thermo scientific, Rockford, IL, USA) was used for protein quantification. After loading equal amounts of protein per lane (30 μg protein), 7 to 20% Protean-TGX (Tris-Glycine eXtended) gels (BIO RAD, Hercules, CA, USA) was electrophoresed at 250 V for 30 to 45 min and transferred to a TransBlot Turbo PVDF (Polyvinylidenfluorid) membrane (BIO RAD) using a TransBlot Turbo system (BIO RAD). Membranes were blocked in EveryBlock Blocking buffer (BIO RAD) according to the manufacturer’s antibody specification protocol for 5 min and incubated overnight at 4 °C with the primary antibodies (AKT rabbit Ab #9272, beta-Actin Rabbit mAB #8457, HSP 90 Rabbit AB #4874, STAT3 rabbit mAB #12640; Cell Signaling Technology, Leiden, The Netherlands) at 1:1000 dilution. Thereafter, the membranes were washed using PBST (phosphate buffered saline + tween 0.025%) and incubated with an Anti-rabbit IgG HRP-linked AB 7074; (1:2000; CST, Denver, MA, USA). Band detection was performed using the ChemiDoc MP imaging system (BIO RAD). Western blotting was performed without replicates. The comparative quantification of Western blot results was carried out using the BIO RAD Image lab software, Version 6.1 (BIO RAD).

## 5. Conclusions

The preliminary data of the present study indicate that GP-2250 has anti-neoplastic effects in BRAF-mutated melanoma cell lines regarding tumor cell viability, proliferation, and apoptosis/necrosis. By contrast, apoptosis/necrosis is not observed in benign primary melanocytes even with the use of the highest concentration. GP-2250 is able to downregulate the gene and protein expression of aberrant tumorigenic pathways in melanoma cell lines. Since GP-2250 is a GAPDH inhibitor, the substance may be a promising combination therapy for tumors presenting the Warburg effect, such as melanoma. Hence, future experiments on a combined treatment using BRAFi/MEKi with GP-2250 in BRAF-mutated melanoma cell lines are warranted. Moreover, it could be intriguing to explore in vivo investigations to complement the findings of the present study.

## Data Availability

The datasets used and analyzed during the current study are available from the corresponding author upon reasonable request.
